# Correlation of ultrasound attenuation with proton density fat fraction across multiple organs in healthy volunteers

**DOI:** 10.1002/mp.70475

**Published:** 2026-05-10

**Authors:** Adrien Rohfritsch, Jules Courgenay, Antoine Biénassis, Elorri Olhagaray, Manon Basso, Benjamin Leporq, Benoit Allignet, David Melodelima

**Affiliations:** ^1^ Laboratory of Therapeutic Applications of Ultrasound INSERM, Centre Léon Bérard Université Lyon 1 Lyon France; ^2^ Univ Lyon, INSA‐Lyon Université Claude Bernard Lyon 1 Villeurbanne France; ^3^ Department of Radiation Oncology Centre Léon Bérard Lyon France

**Keywords:** fat fraction, quantitative MRI, ultrasonic attenuation

## Abstract

**Background:**

Local determination of the attenuation in biological tissues has been the focus of extensive research over the past decades. Its characterization is of major interest for both noninvasive thermal therapies and diagnostic applications. Numerous ultrasound (US)‐based methods have been investigated in recent years to assess attenuation in vivo.

**Purpose:**

In this article, we aim to map US attenuation in four organs of human volunteers: liver, pancreas, kidney, and breast. We propose to compare the mean attenuation values obtained in each tissue with the corresponding proton density fat fraction (PDFF) derived from quantitative magnetic resonance imaging (qMRI). This comparison allows us to (i) present a direct calculation method of the local US attenuation and (ii) investigate the relationship between the average fat content of each organ and its global US attenuation.

**Methods:**

The ultrasonic measurement method is in line with techniques originating from the spectral difference method. Here, the attenuation coefficient (AC) is estimated by insonifying tissues with a single plane wave and acquiring the backscattered echoes. This approach avoids the need for a reference medium to compensate for diffraction and focusing effects. The method is characterized and validated on a calibrated phantom and compared with two commonly used techniques on ex vivo liver tissues. Subsequently, attenuation maps and average values obtained from US imaging in healthy volunteers are compared with PDFF values measured by qMRI. Hepatic (n=12) renal (n=12), pancreatic (n=8) and breast (n=3) tissues were analyzed. Statistical significance was assessed using a paired *t*‐test. To account for multiple comparisons, a Bonferroni correction was applied, resulting in an adjusted 5% significance threshold of p<0.0125. Effect sizes were also reported using Cohen's d parameter. Effect sizes were considered large for d≥0.8.

**Results:**

Measurements on the calibrated phantom showed relative errors between the measured mean values and the manufacturer values of 2% and 9%, respectively. Average AC of each organ was included in the confidence interval of the corresponding literature value. The Pearson correlation coefficient between log(PDFF) and AC slope is $R^{2}=0.73$ (p<0.00025). When each organ was considered separately, no significant correlation was observed between PDFF average values and global US attenuation, as variations between volunteers were found of the same order of magnitude as the standard deviation around each average value.

**Conclusions:**

This work presents an alternative method for in vivo characterization of US attenuation based on the emission of a plane wave, and highlights the impact of fat density on inter‐organ attenuation variations. Together, these results provide new insights into the relationship between tissue microstructure and US attenuation.

## 1 INTRODUCTION

Although ultrasound (US) imaging is a mature medical imaging tool, new techniques are constantly being explored to further improve image quality and extend its clinical usefulness to new applications. In this context, characterizing the elastic and backscattered properties of human tissue has been extensively studied in recent years^1^, ^2^, ^3^ and provides valuable information for diagnosis^4^ or biopathological characterization.^5^ Moreover, US imaging is also widely used to guide treatments performed with high‐intensity focused US (HIFU)^6^ or histotripsy.^7^ In this context, US attenuation is a parameter of paramount interest for both quantifying tissue properties and estimating energy deposition during US treatments. US attenuation can also be used independently to differentiate healthy from pathological tissues.^8^ In the last thirty years, many different experimental setups and signal processing methods have been developed to evaluate US attenuation in the imaging frequency range (i.e., typically between 1 and 20 MHz).^9^, ^10^ Commercial devices recently integrated US attenuation estimators, primarily motivated by the diagnosis and the grading of metabolic dysfunction‐associated fatty liver disease.^11^, ^12^ Recently, efforts have been devoted to developing methods that do not require normalization with reference media.^13^, ^14^ These efforts were mainly driven by the substantial error that occurs when the reference medium differs from the medium being characterized, as discussed in details by Nam *et al.* ^15^ In this context, Rafati *et al* paid particular attention to managing the interfaces between two heterogeneous tissues with different echogenicities.^16^ Other works made use of conventional imaging sequences to characterize the steatosis of murine livers or tissue structural properties.^17^, ^18^

The precise knowledge of tissue attenuation is of great interest for noninvasive thermal therapies such as HIFU since the rate of ablation strongly depends on the attenuation of surrounding and targeted tissues. Based on numerical simulations, Gray *et al* reported a decrease of 43% of the thermally ablated volume in the pancreas when considering the real attenuation value instead of the literature average.^19^ Ritchie *et al.* also quantified the impact of peri‐nephric fat on intensity losses at the focus and on beam focusing during thermal treatment of renal tumors, arguing in favor of a priori aberration correction to bypass the important relative defocusing of the ultrasonic beam.^20^ The recent review by Zulkifli *et al.* on clinical trials dedicated to HIFU ablation of breast tumors reveals substantial variability in the percentage of patients whose entire tumor volume was treated, ranging from 17% to 100%.^21^ In this clinical context, the attenuation variation in breast tissues among patients seems to be an important factor involved in this variability. In the context of liver cancer, Barrere *et al.* showed that the AC slope differed by a factor of two between primary and secondary liver tumors, with approximately 30% intra‐group variation.^22^ These findings further support the necessity of accounting for attenuation variability during the planning phase of local thermal treatments. Therefore, quantitative magnetic resonance imaging (qMRI) measuring fat content could be a relevant addition to plane wave imaging (PWI) to estimate AC. Altogether, these findings demonstrate that precise characterization of tissue mechanical properties is crucial for achieving homogeneous HIFU ablation across patients.

In this article, we aim to evaluate both local and global US attenuation in various organs and breast tissue, and to investigate the relationship between global attenuation values and proton density fat fraction (PDFF) estimated by qMRI.

PDFF estimation and its link with US‐based metrics have been the focus of extensive research over the past years. In the liver, PDFF measurement is mainly used as a noninvasive method for estimating the progression of steatosis.^23^ This characterization is crucial in the early stages of the Metabolic Associated Fatty Liver Disease. The meta‐analysis published by Gu *et al.* highlighted the high diagnosis accuracy of PDFF measurements in assessing the hepatic fat content.^24^ In parallel, more affordable and accessible methods have been investigated to assess liver fat content, among which US imaging stands out. Among other contributions, Timana *et al.* measured attenuation coefficient (AC) and backscattering coefficient to evaluate the progression of steatosis.^17^ De Robertis *et al.* reported the positive correlation between PDFF and US‐derived fat fraction (a parameter also derived from liver backscattering intensity).^25^ Table 1 of Reference [26] summarizes eight commercial systems that already integrate signal processing tools to estimate liver steatosis grade through attenuation‐related parameters. Among them, five tools are supported by performance studies that give four different threshold values for steatosis detection. These variations in thresholds may be due to differences in acquisition parameters and substantial differences in the normalization methods employed. Overall, these studies support that both US attenuation and PDFF are correlated and strongly associated with the presence of fat inclusions in the liver.

**TABLE 1:** Comparison of attenuation values obtained via the method presented in this article and those provided by the manufacturer of the calibrated phantom in two homogeneous parts with different attenuation values and within the inclusions of the phantom.

	Weakly attenuating part	Strongly attenuating part	Inclusions
Measurements (Np/(cm.MHz))	0.079±0.030	0.10±0.030	0.078±0.020
Reference (Np/(cm.MHz))	0.081±0.008	0.11±0.011	0.081±0.008
Abs. error (Np/(cm.MHz))	0.002	0.01	0.003
Relative error (%)	2.5	9.1	3.7
Cohen's d (↑)	0.09 (small)	0.44 (medium)	0.20 (small)

In the pancreas, Idilman *et al.* demonstrated that fat content also increases in patients with MAFLD.^27^ in another context, recent work by Raitano et al. suggested that pancreatic fat accumulation may be an early indicator of the onset of pancreatic ductal adenocarcinoma (PDAC).^28^ This hypothesis is further supported by Hoogenboom et al., who identified fat deposits on CT images up to three years prior to PDAC diagnosis.^29^ Currently, clinical assessment of pancreatic attenuation is performed primarily through qualitative means, by comparing its echogenicity to that of adjacent organs. This approach is naturally subject to variability due to pathological conditions in neighboring structures, as well as probe positioning.^28^ For these reasons, establishing a link between pancreatic fat content and attenuation is a key challenge for diagnostic purposes.

Fat quantification in the kidney is emerging as an important biomarker for numerous conditions, as detailed by Raphael *et al.* ^30^ These conditions include diabetes, obesity, chronic kidney disease, hypertension, and MAFLD. Furthermore, Altay *et al.* demonstrated that PDFF measurement aids in the differentiation of clear cell Renal Cell Carcinoma from other types of renal tumors, with good sensitivity and specificity.^31^

Breast tissue is mainly composed of glandular structures, connective stroma, and adipose tissue. The comparison between PDFF values and ultrasonic attenuation offers insights into the role of adipose tissue in contributing to US attenuation. The impact of histopathological features of normal and pathological breast tissues was already studied by Landini and Sarnelli in 1986.^32^ Hisanaga *et al.* more recently showed a correlation between peritumoral fat density and prognosis in cases of breast carcinoma.^33^ Altogether, these works support the relevance of identifying the histological factors underlying attenuation differences is a key challenge, particularly for the development of targeted thermal therapies involving HIFU.^34^

This paper is organized as follows. The US AC measurement method is first presented. The methods used to estimate AC and PDFF are first described. Then, application of the US method to a calibrated phantom with different ACs is presented and a comparison with other methods from the literature on an ex‐vivo liver sample is made. Finally, local and global AC estimation in the liver, kidney, pancreas and breast tissues of 12 volunteers are presented and linked with corresponding PDFF average value.

## 2 METHODS

### 2.1 US equipment

For PWI, acquisitions were performed via a 5.2 MHz linear probe of 128 elements (ATL L7‐4, Philips, Eindhoven, Netherlands) connected to a Vantage 256 system (Verasonics, Kirkland Washington, USA). Each element has a width of 250 μm and is separated by a distance of 298 μm. The Verasonics Vantage system provided the raw radiofrequency (RF) signals at a sampling frequency of 20.8 MHz. The US sequence consists of a single plane wave with a central frequency of 5.2 MHz. At the center of the imaging plane, this waveform eliminates the need for normalization by a reference medium. In the same spirit as the spectral difference method (SDM), the method extracts the AC from the decay of backscattered energy with depth in a heterogeneous tissue. The SDM and the spectral log difference method (SLDM) are both widely used^15^, ^35^, ^36^ but necessitate the use of a reference medium to normalize the backscattered spectrum.^10^ The backscattered intensity received by the US imaging probe was obtained on the basis of the beamformed RF signals measured by each element k among the 128 elements, as described by Equation 1:

(1) $$I_{r}^{\left(k\right)}={\left|s_{k}\left(z\right)\right|}^{2}$$

where $s_{k}\left(z\right)$ is the beamformed RF signal acquired by the $k^{th}$ element and where $I_{r}^{(}\left(k\right))$ is the corresponding backscattered intensity. Beamforming was performed using the Stolt's f‐k migration method.^37^ Acquisitions with a single element focused transducer were also performed for comparison of the PWI method with SDM and SLDM methods, using another US system (Vevo 770, Visualsonics Inc, Toronto, ON, Canada). The probe RMV710 (Visualsonics Inc, Toronto, ON, Canada) was used in B‐Mode. The geometric focal distance is located 15 mm from the transducer surface. Acquisitions were performed approximately 1 cm below the sample surface. Acquisitions procedure is the same as the one described in.^38^

### 2.2 Computation of the AC via PWI

The local attenuation of an incident plane wave propagating inside a medium made of scatterers randomly distributed inside a host matrix is the sum of the attenuation by scattering ($\alpha_{s}$) and absorption ($\alpha_{a}$), which can be described by Equation (2):

(2) $$\alpha \left(f\right)=\alpha_{s}\left(f\right)+\alpha_{a}\left(f\right)$$

where α is the local attenuation and f is the frequency of the plane wave. The incident plane wave, $p_{i}$, can be described by Equation (3):

(3) $$p_{i}\left(z,k\right)=Ae^{j\omega (z/c_{\phi }-t)}e^{-\alpha f}$$

where z is the depth inside the heterogeneous medium, A is the amplitude of the wave at location z=0, ω is the angular frequency, and $c_{\phi }=1540$ m/s is the soundspeed velocity. In this article, a linear imaging array is used as ultrasonic source, all elements emitting in‐phase pulses. This typical plane wave sequence is altered on one hand by near field interferences caused by the finite size of the piezoelectric components and on the other hand by the elevation focusing induced by the design of the probe itself. Both effects have been quantified through experimental measures and numerical simulations of the pressure field. Near field effects impose a minimal distance z=2 cm beyond which the field can be described with Equation (3) in the imaging plane. Elevation focusing weakly impacts the pressure profile. The width at half maximum of the field in the elevation dimension is of several millimeters. At the scale of soft tissue microstructure (the cellular level), this width is large enough to support the idea that the scatterers are locally excited by a plane wave. Experimental and numerical measures of the emitted field are reported as Supplementary Material. The incident intensity can therefore be described by Equation (4):

(4) $$I_{i}\left(z,f\right)=A^{2}e^{-2\alpha (f)z}$$

Considering the cells as the principal heterogeneities involved in scattering,^38^ the medium is dense at the scale of the wavelength ($\lambda =c_{\phi }/f_{c}=300$ μm). This remark implies that the backscattered field at depth z is composed of scattered fields that are close in phase. For this reason, we can assume that the averaged backscattered intensity ($I_{r}$) at depth z can be described by Equation (5):

(5) $$I_{r}\left(z,f\right)=A^{2}e^{-4\alpha (f)z}$$

Equation (5) is valid under the assumption that scatterers in the medium are small compared to the wavelength λ (typically of radius a≪0.1λ) and that the impedance contrast between the scatterers and the surrounding medium is low (typically lower than 0.1, as reported in the human liver^38^). Together, these two conditions limit the influence of multiple scattering which is supposed negligible in the following. According to Equation (5), it is possible to calculate the attenuation inside the medium, which is only based on the reflected intensity, as described by Equation (6):

(6) $$\alpha_{\mathrm{PWI}}\left(f\right)=-\frac{1}{4A^{2}}\frac{\partial \ln(I_{r}\left(z,f\right))}{\partial z}$$

In many biological tissues, the attenuation α(f) varies almost linearly with frequency.^39^ The attenuation AC will be evaluated under this assumption such that $\alpha_{0}=\alpha_{\mathrm{PWI}}/f$.

### 2.3 Computation of the AC via the SDM and the SLDM

The SDM and SLDM were used to validate the method proposed in this work. To extract the AC using these two methods based on RF signals acquired with a focused transducer, a reference medium is required. In the reference medium, the attenuation ($\alpha_{\mathrm{ref}}$) and the speed of sound are known. For an accurate compensation of the diffraction effects, the speed of sound of the reference medium must be close to that of the medium to be characterized. These methods enable the characterization of local attenuation based on the analysis of the backscattered signal within a homogeneous region of interest (ROI) centered on the focal zone of the transducer. This assumption of homogeneity is directly linked to the previous comments on the validity of Equation (5). That is to say, for an ROI where SDM and SLDM can be applied, PWI method is also applicable. Variations in the power spectral density of the backscattered signal, denoted $S_{s}\left(f,z\right)$ for the unknown tissue and $S_{\text{ref}}\left(f,z\right)$ in the reference medium, as a function of depth z within this ROI, lead to the characterization of attenuation using the SDM method, denoted as $\alpha_{\mathrm{SDM}}$ and given by Equation (7):

(7) $$\begin{array}{cc} \alpha_{\mathrm{SDM}} & =\alpha_{\mathrm{ref}}-\frac{1}{4}\frac{\partial }{\partial z}\left(\ln\left(\frac{S_{s}\left(f,z\right)}{S_{\text{ref}}\left(f,z\right)}\right)\right)\\ & =\alpha_{\mathrm{ref}}-\frac{1}{4}{\tilde{S}}_{z}\left(f\right) \end{array}$$

Considering the SLDM, only proximal ($z=z_{p}$) and distal ($z=z_{d}$) sub‐regions in the ROI are considered, which leads to characterize $\alpha_{\mathrm{SLDM}}\left(f\right)$ as given by Equation (8):

(8) $$\alpha_{\mathrm{SLDM}}=\alpha_{\mathrm{ref}}-\frac{1}{4(z_{p}-z_{d})}\left({\tilde{S}}_{z_{p}}\left(f\right)-{\tilde{S}}_{z_{d}}\left(f\right)\right)$$

Further details on the derivation of Equations (7) and (8) and on their physical assumptions can be found in References.^10^, ^35^ The reference phantom used in this article was provided by the University of Wisconsin (Wisconsin, USA) and consisted of 6g/L of glass beads with radii ranging from 0.4 to 6 μm in a gel‐surrounding medium.

### 2.4 US measures of $\alpha_{0}$ in reference gelatin phantoms, ex‐vivo tissue and healthy volunteers

Attenuation measurements using PWI were first performed in a gelatin calibrated phantom (CIRS 040GSE, CIRS inc, Norfolk, USA). The phantom contained inclusions with different echogenicities, as well as a tissue‐mimicking background, with distinct manufacturer‐reported AC values. One part of the phantom background had an AC slope $\alpha_{0}$ of 0.079±0.006 Np/(cm.MHz), and the other was more attenuating, with a slope of 0.107±0.006 Np/(cm.MHz). Acquisitions were performed at the level of the inclusions (results of section III.A) and between the inclusions, on homogeneous sections (results of section III.B). The US imaging probe was placed at the top of the phantom with a thin layer of US coupling gel. Plane wave images were acquired in the homogenous parts of the phantom as well as along inclusions for which both echogenicity and attenuation change locally. These measures were used to quantify the efficacy of the PWI method in terms of resolution. The absolute error, defined as the difference between the measurement made by the tool and the reference (manufacturer‐reported) value, was calculated according to Equation (9). The relative error, defined as the absolute error divided by the reference value and expressed as a percentage, was calculated according to Equation (10):

(9) $$\begin{array}{cc} \mathrm{Absolute}\mathrm{error} & =|\mathrm{Measured}\mathrm{value}-\mathrm{Reference}\mathrm{value}| \end{array}$$

(10) $$\begin{array}{cc} \mathrm{Relative}\mathrm{error} & =100\times \frac{\mathrm{Absolute}\mathrm{error}}{\mathrm{Reference}\mathrm{value}} \end{array}$$

Then, estimation of averaged AC using PWI method on a freshly excised bovine liver sample was compared to estimations based on SDM and SLDM method. The sample was degassed for 20 min and then immersed in a degassed water tank. RF signal acquisitions using the L7‐4 probe (PWI method) and the RMV710 probe (SDM and SLDM) were performed. It must be noted that the frequency bandwidths of these probes are not the same, which can induce a bias in the estimation of the AC. However, AC nonlinearity in liver tissue is known to be small (see, e.g., Ref. [40] and references therein, or Ref., [41] or also Ref. [38]). Therefore, comparing AC slopes obtained over different bandwidths can be done without inducing large interpretation errors. This justifies comparing the AC slopes obtained from data collected by these two probes. Unpaired two samples *t*‐tests were performed and Cohen's d values were calculated to evaluate the consistency between the predictions of the three methods.

In vivo experiments were finally performed on four organs (breast, liver, pancreas and kidney) of 12 healthy volunteers (8 men, 4 women). To be eligible, volunteers were at least 18 years old, in good clinical condition (World Health Organization performance status 0) and not pregnant. Patients gave informed consent. People were not eligible if they had BMI >30. This limitation was due to the depth of the US images (<6 cm). No other exclusion criteria were imposed in the article. The imaging probe, US scanner, and plane wave sequences were characterized based on hydrophone pressure measurements of the mechanical indices (MI) and spatial peak time average intensity ($I_{\text{spta}}$) for various driving voltage values, up to 50V. The highest values obtained were respectively MI=0.59 and $I_{\text{spta}}=1.21$ mW/${\mathrm{cm}}^{2}$. These values are below the limits set by the Food and Drug Administration (FDA) and European authorities.^42^ In this article, we used driving voltages between 20and 40V, resulting in a maximal values of MI = 0.51 and $I_{\text{spta}}=0.91$ mW/${\mathrm{cm}}^{2}$. Experimental measures of MI and $I_{\text{spta}}$ as well as acoustic field characterization are reported in the Supplementary Material. The volunteers were given an explanation of the article's objectives and risks, and consent was systematically obtained. The acquired US data were anonymized before postprocessing. For both abdominal and breast US scanning, the volunteers were seated in semi‐upright position and scanned by a trained and certified radiologist (M. B.) with more than five years of professional US imaging experience at university hospital Croix Rousse (Lyon, France). The US imaging probe was positioned on the right side of the abdomen so that the liver, the pancreas and the kidney could be viewed via conventional B‐mode imaging in a parasagittal scan plane. For breast tissue, scans were performed on both breasts, positioning the probe successively on the upper and lower parts of the breast. Once the probe was positioned, PWI was used, and RF signals were acquired for postprocessing as described previously. For each volunteer, two acquisitions were performed per organ. In absence of artifacts in the organ on B‐mode image, both acquisitions were postprocessed and AC slopes averaged. These four organs were chosen because they are accessible for US imaging and are also the target for many US‐guided HIFU or focused US treatments.^43^, ^44^, ^45^ Kidney and liver were imaged in twelve volunteers. Among these, the pancreas was visible in only eight cases due to the limited imaging depth accessible with the US probe. Moreover, US attenuation of both organs has been documented ex vivo or in vivo for comparison purposes (see Table 3). Breast tissue was imaged in three volunteers.

**TABLE 2:** Comparison of attenuation values obtained via PWI, SDM, and SLDM methods.

	PWI	SDM	SLDM
average ± std (Np/(cm.MHz))	0.079±0.011	0.082±0.023	0.081±0.021
	PWI vs. SDM	PWI vs. SLDM	SDM vs. SLDM
p−value (↓)	0.913 (ns)	0.636 (ns)	0.813 (ns)
Cohen's d (↑)	0.164 (small)	0.127 (small)	0.0363 (small)

*Note*: Significance levels are p<0.0125 (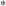), p<0.0025 (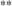) and p<0.00025 (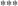); ns: non‐significant (p≥0.0125). The arrows indicate the direction in which the parameter gains significance.

**TABLE 3:** Comparison between the measured AC slope $\alpha_{0}$ in each organ/tissue and values reported in the literature. Relative errors were computed with Equation (10).

	Kidney (n=12)	Liver (n=12)	Pancreas (n=8)	Breast (n=3)
Measured value (average ± std) (Np/(cm.MHz))	0.066±0.007	0.075±0.008	0.098±0.016	0.14±0.014
Literature value (mean [min, max]) (Np/(cm.MHz))	0.060 [0.051, 0.064] Insana *et al.* ^51^	0.068 [0.056, 0.079] Fujii *et al* ^40^	0.15 [0.066, 0.232] Gray *et al* ^19^	0.13 [0.092, 0.17] D'Astous *et al* ^52^
Relative error (mean, [min, max]) (%)	10 [0.0, 29]	10 [0.0, 34]	35 [0.0, 58]	7.7 [0.0, 52.2]

In all the cases, the value of each pixel of the local AC image was obtained via exponential regression on the windowed temporal signal. The window width along the acoustic axis was 5 mm in both directions, which corresponds to approximately 17 wavelengths. In the horizontal direction, the width covers a distance of approximately 15 piezoelectric elements. All measured data are presented as average values ± standard deviations. The global attenuation of each tissue was obtained by averaging the local attenuation values. Segmentation was performed manually based on B‐mode images and validated by a trained radiologist.

### 2.5 MRI acquisition

All the volunteers also underwent MRI acquisitions, performed on a 3T scanner (VIDA system, Siemens Healthineers, Erlangen, Germany) using an 18‐channel matrix breast coils. A 3D spoiled‐multiple echoes gradient echo sequence with a bipolar readout gradient was used. Acquisition parameters were: TR/FA 15ms/5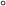; 10 echoes time (TEs) chosen to emphasized fat and water phase (in/out) at 3T (first echo time: 2.4 ms, echo spacing:1.2 ms); a 1860 Hz.pixel‐1 receiver bandwidth; a 25% phase oversampling in the slice direction. Geometric parameters were a 400 by 250 ${\mathrm{mm}}^{2}$ in plane rectangular FOV; a 192 × 120 pixels acquisition matrix with interpolation to 384 × 240 pixels with 40 axial slices of 4 mm thickness giving a resolution of 1.04 × 1.04 × 4 mm^3^. GRAPPA parallel imaging was used with a factor 2. Scan duration was 1 min. Phase and magnitude images were systematically saved.

### 2.6 Proton density fat fraction estimation using quantitative MRI

To quantify PDFF, we used the method described in Leporq *et al.* ^46^ and implemented in Matlab R2023b (The MathWorks Inc., Natick, Massachusetts, USA). Briefly, a specific phase correction algorithm was used to temporally unwrap and correct the native phase images for zero‐ and first‐order phase and rebuild the B0‐demodulated real part images. Then, using a model of fat 1H MR spectrum integrating eight components, fat ($\rho_{f}$) and water ($\rho_{w}$) proton densities were derived and PDFF was computed as follow:

(11) $$\mathrm{PDFF}=\left(\frac{\rho_{f}}{\rho_{f}+\rho_{w}}\right)\times 100$$

**FIGURE 1:**
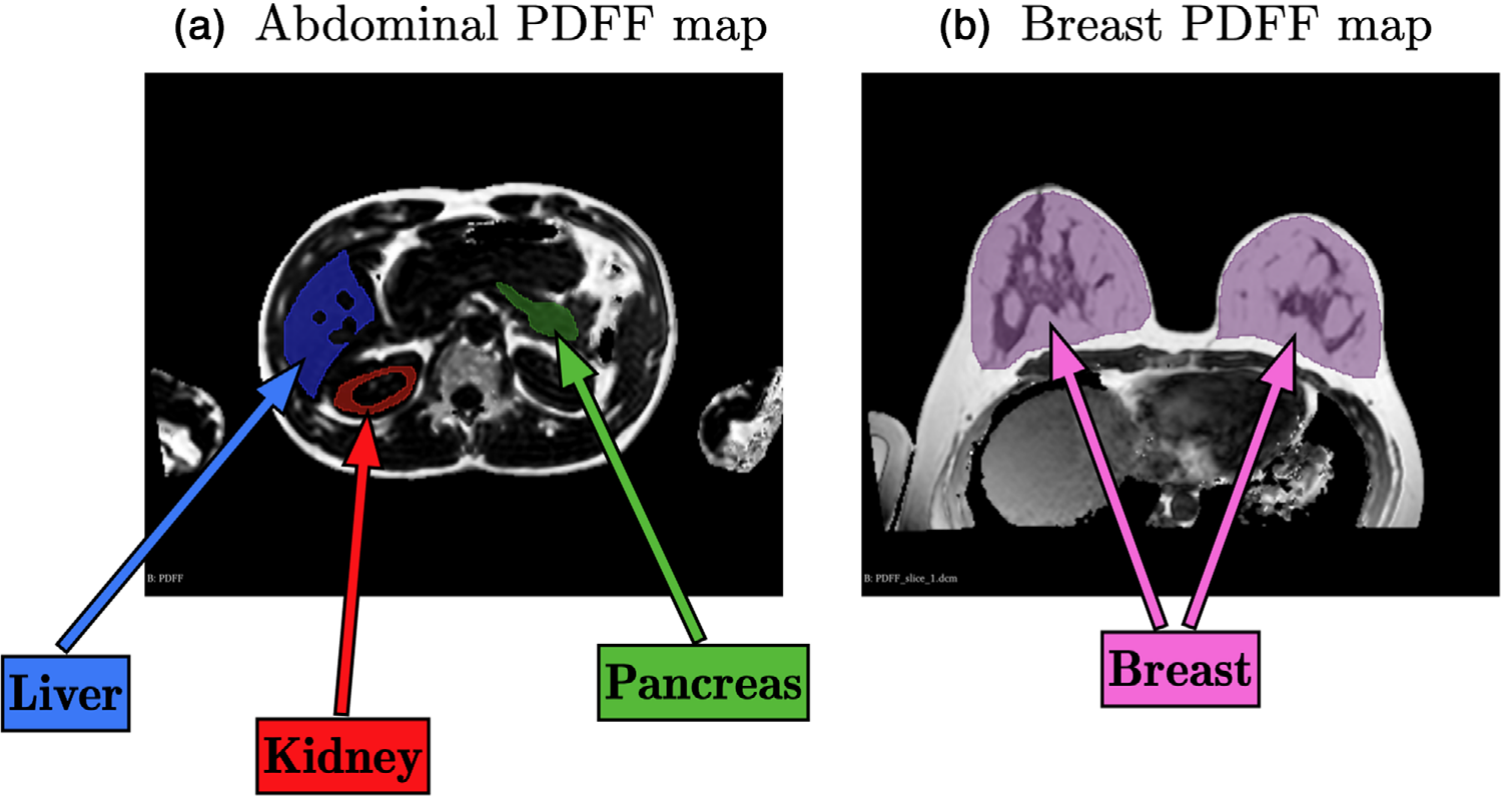
PDFF maps obtained by qMRI in (a) the abdomen of a healthy volunteer and (b) in the breasts of a healthy volunteer. Examples of segmentations of the liver, the kidney and the pancreas are drawn in various colors in the imaging plane. Zero PDFF values were excluded from the analysis.

PDFF maps were segmented in 3D using the 3D Slicer software. The segmentations were validated by a trained radiologist. Figure 1 shows an example of abdominal and breast segmentations. In all tissues, PDFF values equal to zero were excluded from the analysis, as they were assumed to be artefactual. The liver, pancreas, and breast tissue were analyzed in their entirety. For the kidney, only the capsule and cortex, that are located at the periphery, were analyzed. This choice was made to remain consistent with the US measurement, which does not allow AC estimation in the fluid‐filled central region of the organ.

### 2.7 Statistical analysis

Comparison between PWI, SLDM, and SDM methods was carried out through the computation of an unpaired Student t−test. The statistical significance of the linear regression between PDFF and AC values was performed using a paired Student t−test. Cohen's effect size parameter was also calculated as a complementary method to estimate the difference between two groups a and b.^47^ This effect size parameter, d, is given by Equation (12)

(12) $$d=\frac{\bar{X_{a}}-\bar{X_{b}}}{\sqrt{\frac{\sigma_{a}^{2}+\sigma_{b}^{2}}{2}}},$$

with $\left(\bar{X_{i}},\sigma_{i}\right)$ respectively the mean and the standard deviation of group i. According to Cohen, empirical threshold values classify d<0.2 as a small effect size, d=0.5 as a medium effect size, and d>0.8 as a large effect size.^47^

To address the potential increase in statistical type I errors due to multiple comparisons, a Bonferroni correction was applied. Since four statistical tests were performed, the 5% significance threshold was adjusted to α=0.05/4=0.0125, the 1% significance threshold was adjusted to α=0.01/4=0.0025 and the 0.1% significance threshold was adjusted to α=0.01/4=0.00025. Statisticial analysis was performed using GraphPad Prism version 10.4.0 for Windows (GraphPad Software, Boston, Massachusetts USA).

## 3 RESULTS

### 3.1 Attenuation measurements in a reference gelatin phantom containing inclusions

**FIGURE 2:**
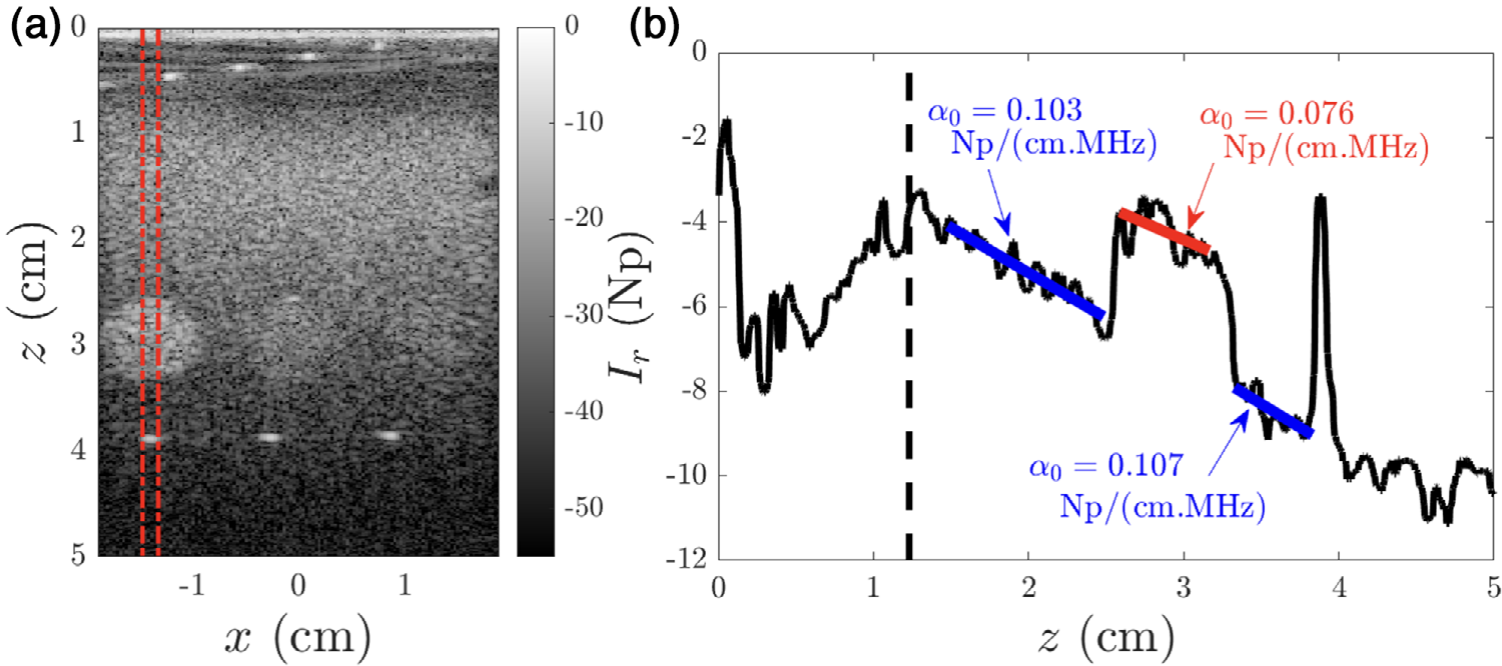
(a) B‐Mode image of the calibrated phantom presenting an attenuation of 0.107±0.006 Np/(cm. MHz) and containing three inclusions with different echogenicities and lower attenuation (0.079±0.006 Np/(cm. MHz)). (b) Backscattered intensity $I_{r}\left(z\right)$ (log. scale) average in the part of the phantom highlighted by the two red dotted lines in (a). The AC measured inside the inclusion and in the surrounding background are reported. The average attenuation slopes measured for the whole phantom are reported in Table 1.

The portion of the CIRS phantom containing inclusions was firstly used to test the relevance of the proposed approach for accurately estimating attenuation in a medium composed of multiple echogenicities and attenuation values. The objective of this first step is not to construct a map of the local AC slope but rather to estimate its value in predetermined regions based on Equation (6). ACs of the calibrated phantom are reported in Table 1. Figure 2a shows the B‐Mode image acquired in the part of the calibrated phantom containing an inclusion immersed in the strongly attenuating part of the phantom. Figure 2b shows the discontinuous backscattered intensity $I_{r}\left(z\right)$ as a function of depth z between the lines shown in Figure 2a. Different echogenicities appear at the depth of the inclusions (2.5 cm <z<3.3 cm), and hyperechoic spots located at approximately z≈3.9 cm are also clearly visible.

In this case, the calculation of the AC must be limited to regions at a distance from the interfaces separating zones of different echogenicities to avoid misestimating the AC. Although the three inclusions measured less than 1 cm (8 mm in diameter), the exponential decay of $I_{r}$ allowed us to estimate the attenuation in all the inclusions. On average, the estimated attenuation in the inclusions was 0.078±0.020 Np/(cm. MHz), which is in agreement with that provided by the manufacturer (Table 1). On the other hand, the spot downstream of this first inclusion is too small to allow precise calculation of the AC, which illustrates one of the limits of the method. Indeed, the spatial resolution can never be smaller than a characteristic length, which is proportional to the extinction mean free path inside the medium and therefore depends on microstructural properties and frequency.^48^ It is worth noting that this limitation also constrains the other methods based on backscattered signal analysis. In the background, the average estimated attenuation was 0.10±0.03 Np/(cm.MHz), which is also close to the value provided by the manufacturer (0.11±0.011 Np/(cm. MHz)). The comparison between the measured values and those provided by the manufacturer highlights effect sizes (Cohen's d, Table 1) ranging from small (for the weakly attenuating region of the phantom) to medium (for the strongly attenuating region).

### 3.2 Local attenuation mapping in reference gelatin phantoms without inclusions

**FIGURE 3:**
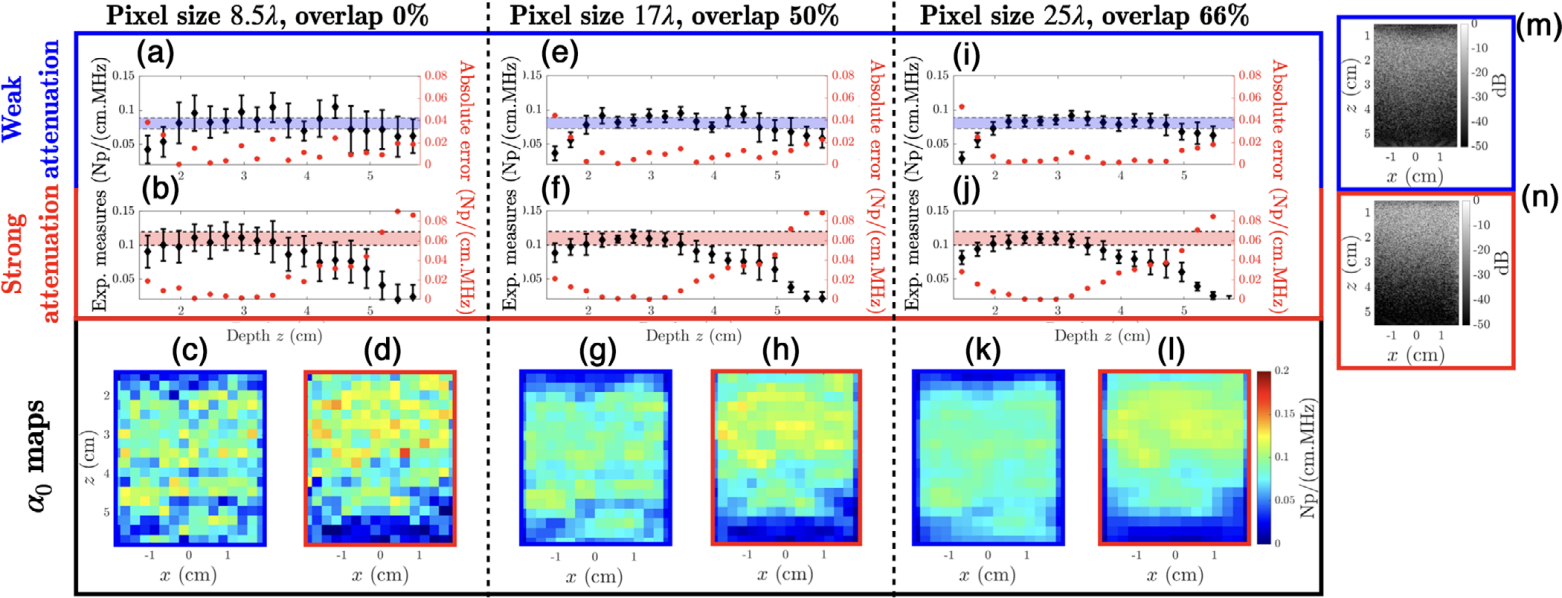
Estimation of the local AC in two parts of the gelatin calibrated phantom using the PWI method. Pixel size varies between 8.5λ (a–d), 17λ (e–h) and 25λ (i–l). In the upper panel are presented estimations of the local AC versus depth z, averaged over the lateral (x) dimension. The black error bars correspond to the variability of the measurement along the lateral dimension. The red points (indexed on the right y‐axis) correspond to the absolute error between the measures and the value provided by the manufacturer. Horizontal shaded areas correspond to the attenuation interval of the manufacturer. In the lower panel are shown the local AC maps for each resolution and each part. (m) and (n) are respectively typical B‐mode image of the weakly attenuating and strongly attenuating parts of the phantom.

To estimate the accuracy of the PWI method in terms of spatial resolution, we calculated α(f) in the imaging plane by estimating this parameter on pixels of various size. Both sections of the calibrated phantom (high and low attenuation values) were used. Figure 3 summarizes the results of this article. Three pixel sizes were considered: 8.5λ (approximately 2.5 mm at 5.2 MHz), 17λ, and 25λ.

On each pixel surface, RF lines were averaged in the lateral (x) dimension and an exponential fit on $I_{r}\left(z\right)$ with depth was then used to estimate the AC. To maintain the same number of pixels in the image plane, the overlap ratio was adjusted with respect to the resolution. Figure 3a, b, e, f, i, j respectively present the mean values of α(f) and the absolute error obtained as a function of depth z for the resolution of 8.5λ, 17λ, and 25λ. The error bars at each depth represent the standard deviation of the estimation along the x−direction. In the low attenuating part of the phantom, the three resolutions considered show good agreement with manufacturer values for depths between 2 and 5.7 cm. Reducing the pixel size increases variability in both spatial dimensions, but the estimation for 8.5λ size pixels remains accurate despite these variations. The maps obtained for each case are shown in Figure 3g–l. For the highly attenuating phantom (Figure 3b, d, f, h, j, l), the estimation becomes less reliable for depths >5 cm than in the lower attenuating phantom (Figure 3a, c, e, g, i, k). This is due to a lower signal‐to‐noise ratio. Therefore, a simple way to address this limitation would be to increase the probe's emission power. A comprehensive article of the method's performance across different heterogeneous media will be the subject of future work. Results presented thereafter were obtained with pixel of size 17λ and an overlap ratio of 95%. Depending on the excitation voltage, the maximal distance was set between 5.5 and 6.5cm from the surface of the probe.

### 3.3 Comparison between PWI, SDM, and SLDM

**FIGURE 4:**
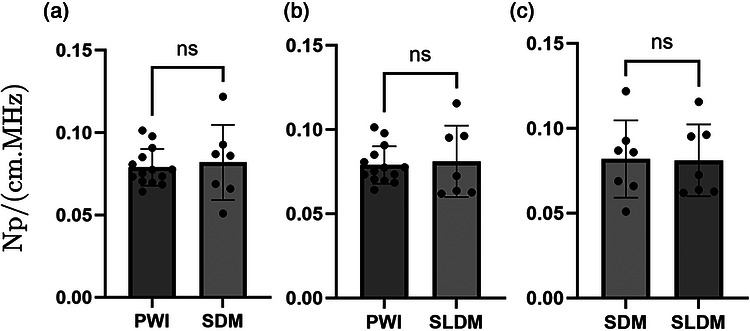
Unpaired Student *t*‐tests between AC slope $\alpha_{0}$ estimated with PWI, SDM, and SLDM methods; ns: non‐significant (p≥0.0125).

ACs measured on freshly excised bovine liver samples using PWI (Equation (6)) and both spectral methods SDM (Equation (7)) and SLDM (Equation (8)) were compared. ACs using PWI were estimated by averaging values across the entire imaging plane, for depth between 2 and 5cm. Results are presented in Figure 4. Average ± standard deviation values, p‐values, and Cohen's d are reported in Table 2. Across all method pairs, p‐values are higher than 0.5 and Cohen's d is consistently lower than 0.2, indicating small effect sizes. The maximal difference of 3.6% is observed between the average values of the PWI and SDM methods, further supporting the practical equivalence of the three methods relative to their respective precision. Altogether, these results endorse the fact that the PWI method allows to efficiently assess local and average AC in soft tissue.

### 3.4 Correlation between average attenuation and PDFF in soft tissues

**FIGURE 5:**
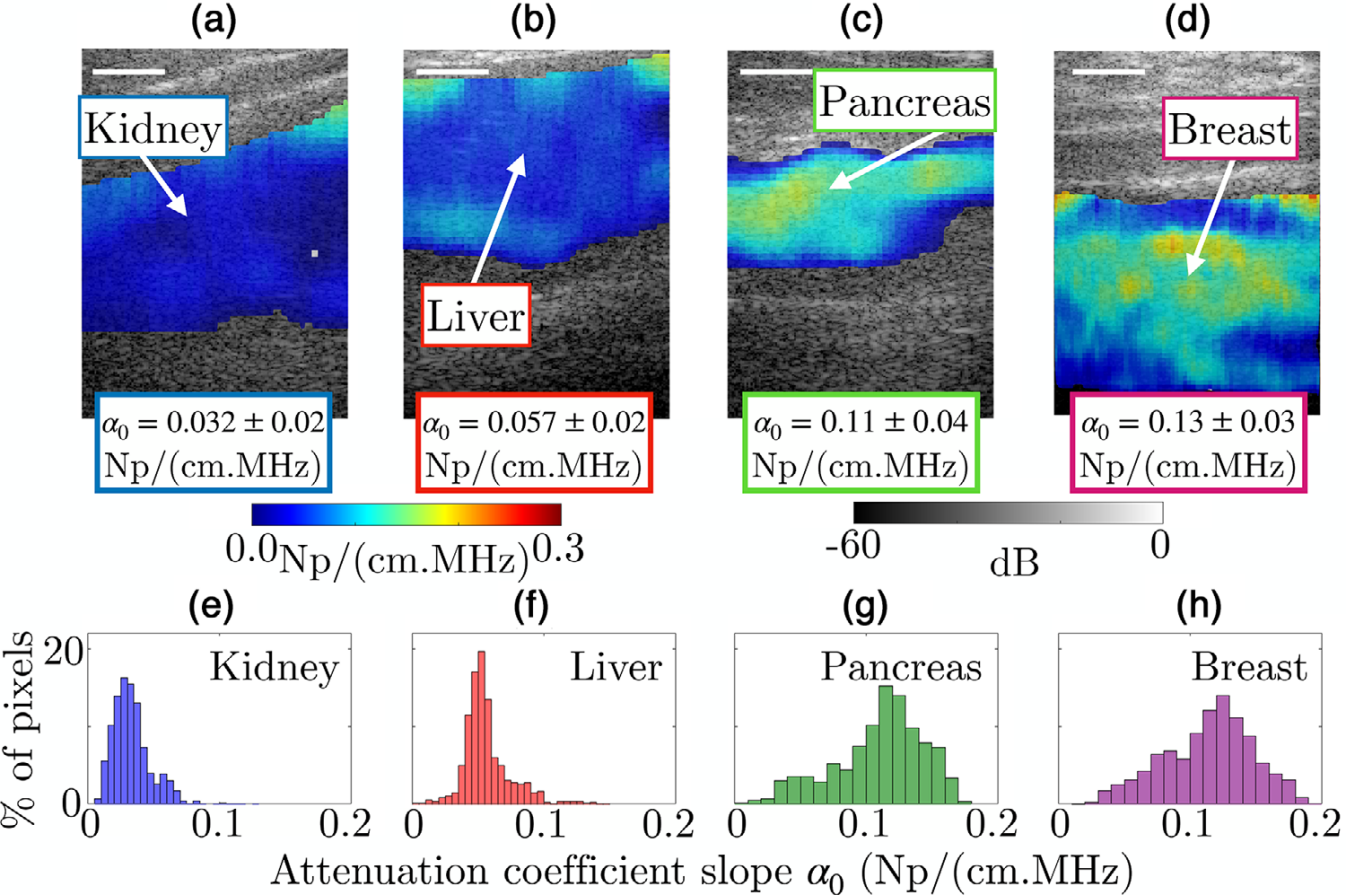
AC maps obtained with PWI method, superimposed on corresponding B‐mode images of (a) kidney, (b) liver, (c) pancreas and (d) breast tissues of healthy volunteers. The histograms corresponding to the AC values of maps (a)–(d) are shown in (e)–(h), respectively.

**FIGURE 6:**
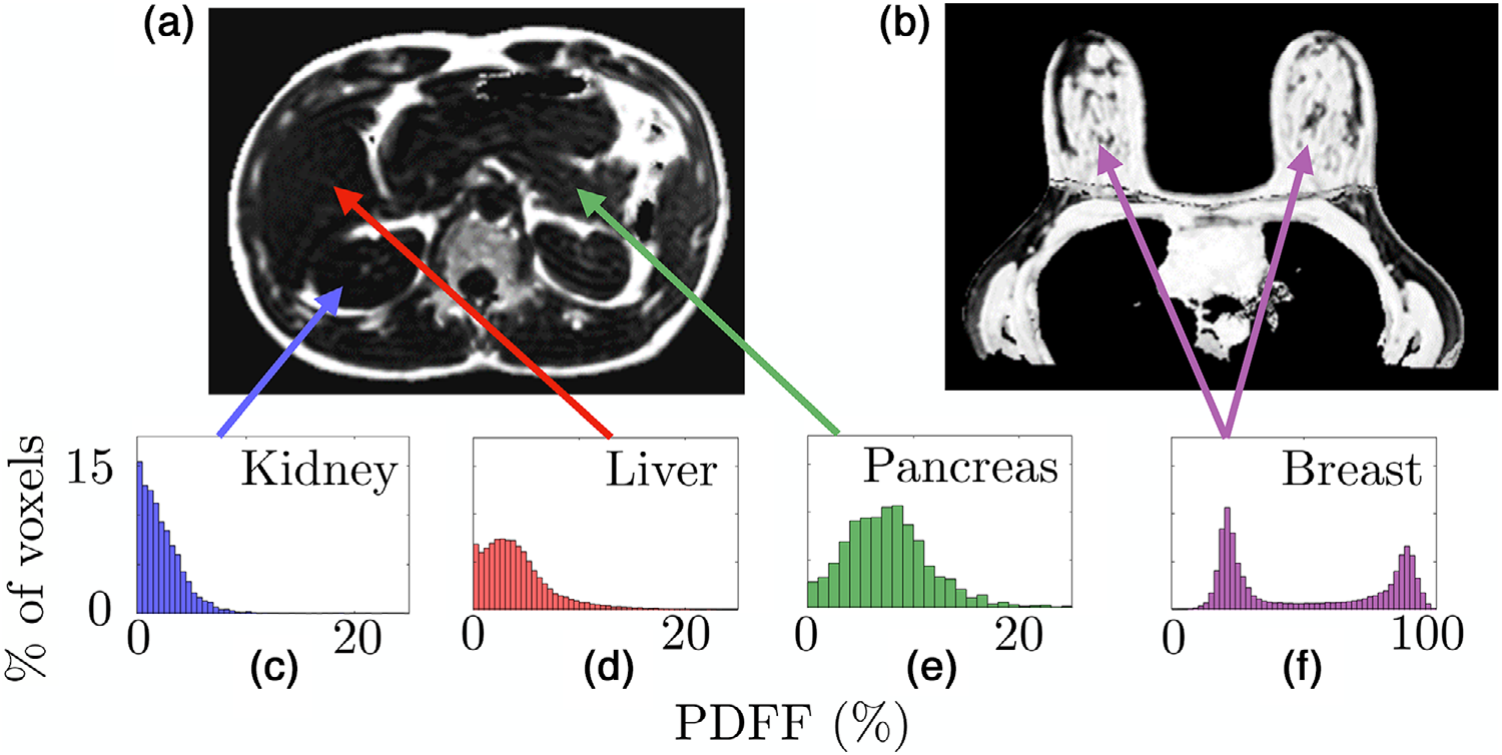
PDFF maps obtained on (a) abdominal and (b) breast qMRI acquisitions of two healthy volunteers. Corresponding PDFF histogram of voxel values of the whole organ on (c) liver, (d) kidney, (e) pancreas and (f) breast tissues.

**FIGURE 7:**
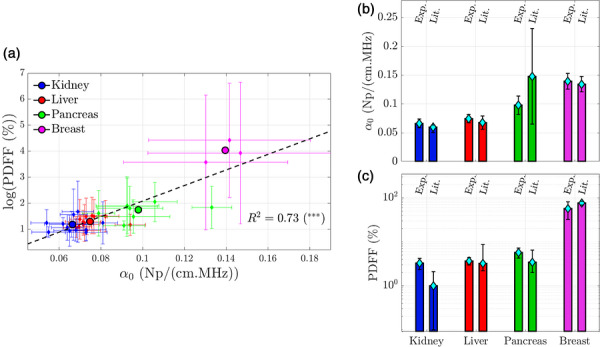
(a) PDFF average values versus average AC slope $\alpha_{0}$. Small diamond markers: average value for each healthy volunteer. Large circular marker: average value across all volunteers. (b) Experimental average AC slope $\alpha_{0}$ compared to literature values for each tissue. References are given in Table 3. (c) Experimental average PDFF compared to reference values for each tissue. References are given in Table 4.

Representative AC and PDFF maps and corresponding histograms obtained respectively via the PWI method and qMRI acquisitions in healthy volunteers are shown in Figures 5 and 6. Figure 7a shows all PDFF values (log scale) as a function of $\alpha_{0}$ for all studied tissues. This figure provides a clear visualization of the overall trend: the higher the fat content in the tissue, the greater the US attenuation. The Pearson correlation coefficient between log(PDFF) and $\alpha_{0}$ is $R^{2}=0.73$ among all the organs. The associated p−value indicates high significance (p<0.00025). This key result supports the conclusion that PDFF is a relevant parameter for distinguishing large attenuation differences. Average and standard deviation values of average AC and average PDFF are respectively reported in Tables 3 and 4.

**TABLE 4:** Comparison between the measured PDFFs in each organ/tissue and values reported in the literature. Relative errors were computed with Equation (10).

	Kidney (n=12)	Liver (n=12)	Pancreas (n=8)	Breast (n=3)
Measured value (mean/IQR) (%)	3.2/3.1	3.7/3.4	5.7/5.3	57/37
Literature value (mean [min, max]) (%)	1.0 [0.90, 1.1] Table 3 (Control) from Liu *et al* ^53^	3.2 [0, 6.4] Table 1 from Rodge *et al.* ^54^	3.4 [2.0, 6.5] Figure 5b from Fukui *et al* ^55^	76 [58, 93] Table 2 and text from Borde *et al* ^56^
Relative error (mean, [min, max]) (%)	220 [190, 255]	16 [0, 42]	67 [0, 185]	26 [2.3, 39]

As shown in Figure 7b, the mean values of $\alpha_{0}$ obtained experimentally are consistent with those reported in the literature: the relative difference between the experimental mean and the literature value ranges from 0% to 35%, and for each tissue, the experimental values lie within the confidence interval of the corresponding literature value. In the case of breast tissue, d'Astous *et al.* distinguish between fat and parenchyma and report two separate values. In our article, we assumed that breast tissue was composed of 55% fat and 45% of fibroglandular tissues to estimate the reference AC value (Table 3). This is an arbitrary but age‐consistent assumption (volunteers being all under 30 years old).^49^ The choice to include only people within a single age group is motivated by the well‐known strong dependence of breast structure on age.^50^ To minimize this variability–which lies beyond the scope of the present article–only this age category was considered. It is worth noting that the standard deviations in pancreatic and breast tissues of each volunteer are higher compared to those in the liver and kidney. This should be interpreted in parallel with the stronger scattering properties of the breast and pancreas, which lead to greater local uncertainties in AC estimation.

Table 4 and Figure 7c summarize the mean PDFF values obtained for each tissue, along with the interquartile range (IQR), and compare them to values reported in the literature. For the liver, kidney, and pancreas, the measured values are in good agreement with the literature. In the case of the kidney, the very low fat content leads to relatively large relative error (ranging from 190% to 255%), although both the experimental mean (3.2%) and the reference value (1%) remain of the same order of magnitude. In breast tissue, the mean PDFF values obtained are generally lower than those reported in the literature. This discrepancy may be partly explained by the young age of the volunteers (≤30 years) and their small number, compared to larger, more representative population‐based studies.

## 4 DISCUSSION

The method developed in this work to extract the AC relies on the decay of the time‐domain signal with depth. The use of a single plane wave in emission eliminates the need for a reference medium, as diffraction and in‐plane focusing effects can be neglected when positioned sufficiently far from the probe surface. Moreover, the MI and the $I_{\text{spta}}$ are also weaker compared to conventional US imaging sequence. However, this comes at the cost of a low SNR ratio and poor‐quality anatomical images. This method was validated on a calibrated phantom and compared with the predictions of two other methods on ex‐vivo tissue. Relationship between fat content in tissues and attenuation was then investigated. In this purpose, attenuation and PDFF measurements were carried out and compared across four different tissues in healthy volunteers. For the four organs examined, the obtained ACs were consistent with values reported in the literature. Regarding the pancreas, the 22% difference observed between the mean value obtained in this article and the one reported by Gray et al. should be interpreted with caution. Indeed, their work reports variability ranging from single to fourfold across a cohort of 27 patients. The standard deviation around their $\alpha_{0}$ value reaches 49.2%, along with large variations in AC nonlinearity. This highlights the substantial inter‐individual variability of tissue properties, which lies at the core of the motivation for their and our studies. Even though the average value obtained in our work falls within their confidence interval, the number of volunteers and patients included in both studies is not large enough to allow a statistical conclusion. Since inter‐individual variations were small, no significant correlation was observed between PDFF and attenuation within each organ individually. Among all organs, a significant positive correlation ($R^{2}=0.73,p<0.00025$) was observed. This key result supports a strong relationship between fat content and US attenuation.

Several limitations of this article must be acknowledged. First, the current US method does not allow reliable AC measurements near organ interfaces or within regions of interest with large variations in echogenicity. However, this limitation is not inherent to the method itself. As shown in Figure 2, measurement within an inclusion is feasible, provided that the interfaces are well detected. Therefore, prior segmentation and post‐processing improvements could enable the generation of attenuation maps within such inclusions. This challenge has notably motivated the work of Rafati *et al.*, who recently proposed a phantom‐free method for mapping AC within liver tumors.^16^ One limitation of this method lies in the fact that it has so far only been applied to ROIs that are relatively small compared to the size of the organ, which is not the case for the approach proposed here. A comparison of the two approaches will be the subject of future studies.

Secondly, the soundspeed in each organ was assumed constant ($c_{\phi }=1540$ m/s). This directly affects AC estimation, which depends on depth variation derived from velocity (Equation (6)). The resulting error in AC is proportional to the soundspeed error. Estimating this quantity in each tissue using the matrix imaging method^57^ could be performed as an initial step of the characterization process to reduce this source of error.

Also, only average attenuation and PDFF values could be compared. Incorporating anatomical landmarks to extract PDFF from the same region probed by US imaging would allow for a more accurate correlation between fat fraction and attenuation within the same anatomical section.

Additionally, acquisitions were limited to healthy volunteers with BMI <30, and each attenuation and PDFF average values for each organ were tightly clustered around the global mean. This likely explains the absence of a significant correlation between the two quantities within individual organs. Thus, this lack of correlation is not related to the method itself, but rather to the limited variability in fat content. In a clinical context, it is important to understand tissue variability in a healthy population in order to better interpret pathology‐induced changes. Similar trends were reported at low PDFFs by De Robertis *et al.* based on backscatter measurements (Figure 4 of Ref. [25]), as well as by Nishimura *et al.* using the controlled attenuation parameter (CAP) measured with a FibroScan device and the attenuation imaging (ATI) tool developed by Canon Medical Systems (Otawara, Japan) (Figure 2 of Ref [58]).

Finally, it is also important to note that in vivo measurements on healthy organs can be affected by inter‐subject anatomical variability. Indeed, the same organ may be located at different depths and present varying volumes, among other factors. Increased depth leads to a stronger impact of aberrations related to the previously mentioned sound‐speed mismatches and to the increase of multiple scattering effects. An insufficient organ volume prevents reliable estimation of the attenuation decay slope required for AC calculation. In addition, the echogenicity of intermediate tissues may vary, generating reflections that disturb the backscattered signal and hinder accurate measurement. For repeated measurements at a fixed location, motion of subcutaneous tissues and organs may impair reproducibility in the absence of reliable anatomical landmarks or motion correction strategies. To overcome these limitations, an ongoing article is being conducted on ex vivo surgical samples of healthy and tumoral biological tissues, aiming to compare AC estimated using the PWI method with AC measured using the standard pulse‐echo technique.^22^ This comparison, complementary to the results presented here, will enable future clinical studies on diseased patients, with the aim of adapting HIFU treatments and diagnosing fat‐related pathologies.

## 5 CONCLUSION

A new method for estimating the US AC based on PWI was proposed. It was applied to the characterization of four biological tissues, and correlation with PDFF obtained through qMRI highlighted the strong influence of fat content on the average AC slope. The results demonstrate, within the same cohort of volunteers, a positive significant correlation between fat content and global US attenuation across four structurally distinct organs. These findings open new perspectives for biopathological diagnosis with US and may contribute to improving the planning of local thermal therapies.

## AUTHOR CONTRIBUTION

Adrien Rohfritsch designed the research, acquired data, analyzed the data. Jules Courgenay, Antoine Biénassis and Elorri Olhagaray acquired and analyzed the data. Manon Basso performed the US acquisitions. Benjamin Leporq and Benoit Allignet performed the qMRI acquisitions. David Melodelima designed and funded the research. All the authors wrote the paper.

## FUNDING INFORMATION

This project, accredited by Lyonbiopôle, has been carried out thanks to the support of the Cancéropôle CLARA, the Auvergne‐Rhône‐Alpes region and the metropole of Lyon as part of the Proof of Concept program. It was also supported in part by the FUS foundation and by the SIRIC Lyrican under Grant INCa‐DGOS‐INSERM‐ITMO cancer_18003. It also obtained financial support from ITMO Cancer of Aviesan within the framework of the 2021‐2030 Cancer Control Strategy, on funds administered by Inserm.

## CONFLICT OF INTEREST STATEMENT

The authors declare no conflicts of interest.

## Figures and Tables

**FIGURE A1:**
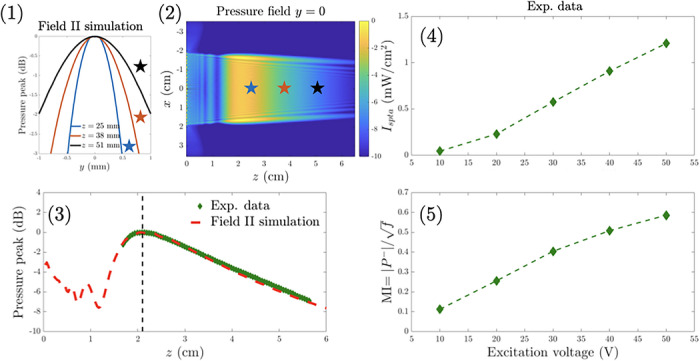
Emission characterization and safety consideration of the L7‐4 probe. (a) elevation scans of the emitted pressure field at different depth z, in the plane x=0 and (b) pressure field in the (x,z) plane. (c) Comparison between numerical scan and experimental measures along the z‐axis in the axis (x=0,y=0) for an excitation voltage of 14V. (d) MI and (e) $I_{\text{spta}}$ measured at the maximum of pressure along the z‐axis for various excitation voltage.

## APPENDIX A APPENDICES

Numerical simulation of the emitted field was performed using Field II software.^59^, ^60^ The imaging probe is the probe ATL L7‐4 (Philips, Eindhoven, The Netherlands), also used for experimental results reported in the present paper. 1D elevation scans are reported in Figure A1a, at three distances *z* from the surface of the probe. 2D scan of the field in the (*x,z*) imaging plane is presented in Figure A1b, and a comparison between numerical and experimental measures of the pressure along the *z* axis is reported in A1c, for an excitation voltage of 14V, demonstrating the quantitative agreement between both. To ensure the safety of imaging acquisitions on healthy volunteers, we characterized the mechanical index and the spatial‐peak temporal average intensity ($I_{\mathrm{spta}}$) at different excitation voltages (Figure A1d and A1e). For both quantities, the maximal value is below the limits set by the FDA and European authorities.^42^
